# Vitamin D deficiency in undifferentiated connective tissue disease

**DOI:** 10.1186/ar2533

**Published:** 2008-10-18

**Authors:** Eva Zold, Peter Szodoray, Janos Gaal, János Kappelmayer, Laszlo Csathy, Edit Gyimesi, Margit Zeher, Gyula Szegedi, Edit Bodolay

**Affiliations:** 1 Division of Clinical Immunology, 3rd Department of Medicine, Medical and Health Science Center, University of Debrecen, Moricz Zs. Str. 22, Debrecen, 4032, Hungary; 2 Department of Rheumatology, Kenézy County Hospital, Bartok Bela Str. 4, 4043, Debrecen, Hungary; 3 Department of Clinical Biochemistry and Molecular Pathology, Medical and Health Science Center, University of Debrecen, Moricz Zs. Str. 22, Debrecen, 4032, Hungary

## Abstract

**Introduction:**

Both experimental and clinical data provide evidence that vitamin D is one of those important environmental factors that can increase the prevalence of certain autoimmune diseases such as systemic lupus erythematosus, rheumatoid arthritis, insulin-dependent diabetes mellitus, and inflammatory bowel disease. The aim of the present study was to investigate the prevalence of vitamin D insufficiency in patients with undifferentiated connective tissue disease (UCTD).

**Methods:**

Plasma 25(OH)D_3 _levels in 161 UCTD patients were measured in both summer and winter periods. Autoantibody profiles (antinuclear antibody, anti-U1-ribonucleoprotein, anti-SSA, anti-SSB, anti-Jo1, anti-Scl70, anti-double-stranded DNA, anti-centromere, anti-cardiolipin, rheumatoid factor, and anti-cyclic citrullinated peptide) and clinical symptoms of the patients were assessed.

**Results:**

Plasma levels of 25(OH)D_3 _in UCTD patients were significantly lower compared with controls in both summer and winter periods (UCTD summer: 33 ± 13.4 ng/mL versus control: 39.9 ± 11.7 ng/mL, *P *= 0.01; UCTD winter: 27.8 ± 12.48 ng/mL versus control: 37.8 ± 12.3 ng/mL, *P *= 0.0001). The presence of dermatological symptoms (photosensitivity, erythema, and chronic discoid rash) and pleuritis was associated with low levels of vitamin D. During the average follow-up period of 2.3 years, 35 out of 161 patients (21.7%) with UCTD further developed into well-established connective tissue disease (CTD). Patients who progressed into CTDs had lower vitamin D levels than those who remained in the UCTD stage (vitamin D levels: CTD: 14.7 ± 6.45 ng/mL versus UCTD: 33.0 ± 13.4 ng/mL, *P *= 0.0001).

**Conclusions:**

In patients with UCTD, a seasonal variance in levels of 25(OH)D_3 _was identified and showed that these levels were significantly lower than in controls during the corresponding seasons. Our results suggest that vitamin D deficiency in UCTD patients may play a role in the subsequent progression into well-defined CTDs.

## Introduction

Environmental factors play an important role in the development and progression of systemic autoimmune diseases along with susceptible genetic and hormonal background. It has been suggested recently that vitamin D is an environmental factor that, by modulating the immune system, affects the prevalence of autoimmune syndromes. Thus, vitamin D deficiency may have a role in the pathogenesis of systemic autoimmune diseases.

The classic and well-known function of vitamin D is to regulate mineral homeostasis and thus bone formation and resorption. On the other hand, a less traditional function of vitamin D has been demonstrated, including substantial effects on the regulation of cell proliferation and differentiation. Also, vitamin D has been described to modulate immune responses [1-6].

Active vitamin D has been shown to inhibit the differentiation and maturation of myeloid dendritic cells to reduce the expression of major histocompatibility complex II, co-stimulatory molecules (CD80, CD86, and CD40), and the maturation proteins (CD1a and CD83) [7]. In addition, the antigen-presenting capacity of macrophages and dendritic cells is suppressed and the immune stimulatory interleukin-12 (IL-12) is inhibited by active vitamin D [8]. Th1 and Th2 cells are direct targets of active vitamin D. Vitamin 1,25(OH)_2_D_3 _decreased the proliferation of Th1 cells and also inhibited the production of IL-2, interferon-gamma (IFN-γ), and tumor necrosis factor-alpha of Th1 cells and had an anti-proliferative effect [3,9]. Furthermore, vitamin D silences the Th17 response and also repairs the number and function of the CD4^+^/CD25^+ ^regulatory T cells, which may prevent the development of autoimmune diseases [9,10]. These findings suggest that the effect of vitamin D is predominantly tolerogenic.

Cantorna and Mahon [11] have shown that vitamin D status as an environmental factor affects autoimmune disease prevalence. The determination of the exact connection is difficult because of the complexity of the vitamin D regulatory system. Moreover, complicated interactions could occur between genes that may affect autoimmune disease susceptibility [11]. Serum levels of vitamin D were significantly lower in systemic lupus erythematosus (SLE) and insulin-dependent diabetes mellitus (IDDM) than in the healthy population [12-15]. Recently, it was also found that lower levels of vitamin D were associated with higher disease activity in rheumatoid arthritis (RA) [6]. An inverse correlation has been described between the supplementation of vitamin D and the development of IDDM and multiple sclerosis (MS) [1,12].

The evolution of diseases with an immune-pathogenetic background is usually slow and progressive. The term 'undifferentiated connective tissue disease' (UCTD) has been used since 1980 to describe a group of connective tissue diseases (CTDs) that lack the characteristics of any distinctive disease. There is great deal of information available regarding the clinical and serological profile of UCTD and the rate of evolution into well-defined CTD [16-18]. About 30% to 40% of patients with UCTD will evolve to defined CTD during the years of follow-up. The higher rate of disease evolution can be seen mostly between the first and second years [18,19]. UCTD has specific signs and/or autoantibodies that are characteristic of autoimmune disease. Mosca and colleagues [18,19] and our previous studies [16] reported that the most frequent clinical manifestations of UCTD were polyarthralgy/polyarthritis, Raynaud phenomenon, serositis (pleuritis and pericarditis), photosensitive rash, xerostomia, and xerophthalmia as well as central nervous system involvement. During the follow-up period, new organ manifestations can appear and the existing clinical and immunological abnormalities can increase in intensity or even become permanent. Evolution to SLE and other systemic autoimmune diseases (mixed connective tissue disease [MCTD], systemic sclerosis, Sjögren syndrome, polymyositis/dermatomyositis, RA, and systemic vasculitis) has also been described.

Until now, there have been no data on the 25(OH)D_3 _levels in UCTD patients. The aim of our study was to assess the vitamin D deficiency in UCTD patients and compare it with controls. We examined the seasonal variance of the vitamin D levels during the summer and winter months. We also determined a possible connection between low levels of vitamin D and the clinical and serological manifestations of the disease. In addition, we determined the prevalence of the 25(OH)D_3 _levels in patients with UCTD and assessed its probable pathogenic role in the progression toward a well-defined CTD.

## Materials and methods

The study population involved 161 patients (154 women and 7 men) with UCTD followed up with and treated at the Division of Clinical Immunology, 3rd Department of Internal Medicine, Medical and Health Science Center, University of Debrecen (Debrecen, Hungary). All patients with UCTD were enrolled based on the following criteria: (a) symptoms and signs suggestive of a CTD not fitting the accepted classification criteria for any of the defined CTDs, (b) disease duration of at least 1 year, and (c) the presence of at least one non-organ-specific autoantibody. No patients had received corticosteroids or immune-suppressive or cytotoxic drugs. Patients with a defined CTD were diagnosed according to the corresponding American College of Rheumatology classification criteria [17,18,20-25]. An informed consent form was signed by all patients approved by the ethics committee of the University of Debrecen. Age- and gender-matched healthy individuals served as controls with no autoimmune/endocrine or malignant neoplastic diseases (residents and health care workers). Clinical data, including age, body mass index, age at diagnosis, and therapy, were obtained by a questionnaire or from patient charts. All patients were followed up with every 4 months. Diagnostic procedures for all patients included chest x-ray, spirometry/diffusion capacity, Doppler echocardiography and high-resolution computed tomography, Schirmer test, sialometry, and radionuclide esophageal transit scingtigraphy. Laboratory measurements included erythrocyte sedimentation rate, full blood count, urine analysis, serum creatine phosphokinase, serum calcium, kidney and liver function tests, thyroid-stimulating hormone, and parathyroid hormone (PTH). PTH was measured using an Advia Centaur autoanalyser (Siemens Healthcare Diagnostics, Deerfield, IL, USA) using reagents and protocols provided by the manufacturer. At the time of study, all patients with UCTD underwent bone densitometry using a Lunar-DPX-L DEXA instrument (Lunar Radiation Corporation, Madison, WI, USA). Bone mineral density (BMD) of lumbar 2 to 4 vertebrae and femoral neck was assessed and T scores were determined. Osteoporosis or least osteopenia was according to the World Health Organization classification criteria (T score of less than -1) [26]. All patients and control subjects had normal mean BMD values. Patients had no signs of renal insufficiency and did not take any vitamin D supplements prior to or in parallel with the investigations. At diagnosis of UCTD, the plasma 25(OH)D_3 _levels were measured. We assessed the plasma 25(OH)D_3 _levels of 161 UCTD patients and 59 control subjects during the summer (from June to October) and winter (from January to May) periods.

### Immune serological analyses

Antinuclear antibodies were determined by indirect immunofluorescence on HEp-2 cells. Anti-U1-ribonucleoprotein (anti-U1-RNP), anti-Sm, anti-SSA, anti-SSB, anti-Jo1, anti-Scl70, and anti-cardiolipin (anti-CL) antibodies were analyzed in all patients by enzyme-linked immunoabsorbent assay (ELISA) in accordance with the instructions of the manufacturers (Pharmacia & Upjohn Diagnostics GmbH, Freiburg, Germany, and Cogent Diagnostics Ltd, Edinburgh, UK). IgM rheumatoid factor (RF) was assessed by nephelometry, and values greater than 50 U/L were considered positive. Anti-cyclic citrullinated peptide (anti-CCP) levels were measured using a second-generation ELISA (Quanta Lite™, CCP ELISA; Inova Diagnostics, Inc., San Diego, CA, USA) and using synthetic citrullinated peptides bound to the surface of a microtiter plate as antigen. The test was performed in accordance with the manufacturer's instructions. Serum samples, collected immediately after the initial diagnosis of patients, were separated and stored at -70°C.

### Determination of vitamin D levels

Plasma levels of 25(OH)D_3 _vitamin of patients and controls were assessed at the Department of Clinical Biochemistry and Molecular Pathology Laboratory of the University of Debrecen Medical and Health Science Center. Samples were analyzed by a high-performance liquid chromatograph (HPLC) method using the Jasco HPLC system (Jasco, Inc., Easton, MD, USA) and a Bio-Rad reagents kit (Bio-Rad Laboratories, Inc., Hercules, CA, USA). The sample (500 μL of plasma from EDTA [ethylenediaminetetraacetic acid] anticoagulated blood) was purified from proteins, and 50 μL of the cleaned supernatant was injected into the instrument. Separation was achieved with a reverse-phase C18 Bio-Rad column (90 × 3.2 mm) (Bio-Rad Laboratories, Inc.). The mobile phase (methanol-water mixture) had a flow rate of 1.1 mL/minute. For quantitative determination of the separated compound, a diode array detector (set at 265 nm) was used. According to current recommendations, plasma 25(OH)D_3 _levels of less than 30 and 10 ng/mL were defined as vitamin D insufficiency and vitamin D deficiency, respectively [27-29].

### Statistical analysis

Data were presented as a percentage or a mean value ± standard deviation. GraphPad Software (GraphPad Software, Inc., San Diego, CA, USA) was used in data interpretation (two-tailed *t *test, chi-square test, and Fisher exact test, logistic regression). A Pierce regression coefficient assay was also performed when required. Multiple linear regression models were used to examine the relationship between vitamin D level clinical signs, smoking, and seasonality. *P *values of less than 0.05 were considered to be statistically significant.

## Results

### Clinical and serological data of 161 patients with undifferentiated connective tissue disease

The mean age at diagnosis of 161 patients with UCTD was 44.91 ± 12.7 years (women/men: 22:1). The mean duration of symptoms at the time of enrollment into the study was 4.09 ± 2.36 years. The ratio of women and men was very similar in the two groups (control group number: 59; mean age: 43.9 ± 15.1 years; women/men: 18:1). There was no difference between the body weight, height, body mass index, and BMD values in patients and controls. The most frequent clinical manifestation of UCTD was polyarthritis (28.5%). The frequency of skin lesions (photosensitivity, erythema, and lymphocytic vasculitis) was 22.9%, and the frequency of Raynaud phenomenon was 17.3%. Xerophthalmia was observed in 15.5% of patients. Pleuritis (5.59%), neuropathy (4.96%), deep vein thrombosis (2.48%), myositis (1.2%), and pulmonal manifestations (1.2%) were less frequent among the first clinical symptoms of the patients. The prevalence of dysmotility was 13.6% in UCTD patients. The most frequent immune serological abnormality in the serum of patients was the presence of anti-nuclear antibody, which was found in 64.59% of patients. The earliest antibody at the onset of UCTD was anti-SSA, which was present in 43 patients (26.7%). Anti-CL autoantibodies could be detected in 40 patients (24.8%). Anti-U1-RNP antibodies were found in the sera of 29 patients (18.0%), anti-Sm antibody in 8 (4.9%), anti-CCP in 14 (8.6%), anti-double-stranded DNA (anti-dsDNA) in 12 (7.4%), anti-SSB in 9 (5.59%), anti-neutrophil cytoplasmic antibody in 4 (2.48%), and IgM RF in 2 patients (1.2%) at the initial diagnosis of UCTD.

### Levels of vitamin D in patients with undifferentiated connective tissue disease

**Figure 1:**
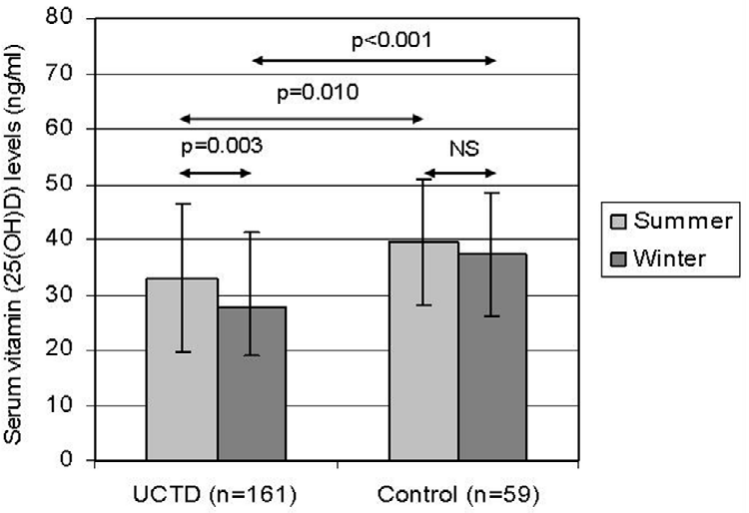
Comparison of vitamin D of undifferentiated connective tissue disease (UCTD) patients with healthy controls during the summer and winter months. NS, not significant.

The summer and winter levels of 25(OH)D_3 _in patients with UCTD were significantly lower compared with healthy individuals (UCTD summer: 33.0 ± 13.4 ng/mL versus control: 39.9 ± 11.7 ng/mL, *P *= 0.010; UCTD winter: 27.8 ± 12.48 ng/mL versus control: 37.8 ± 12.3 ng/mL, *P *= 0.0001) (Figure 1). In UCTD patients, the winter levels of vitamin D were considerably lower than the summer levels (UCTD summer: 33.0 ± 13.4 ng/mL, UCTD winter: 27.8 ± 12.48 ng/mL, *P *= 0.001). In the control group, vitamin D levels were lower in the winter than in the summer, but the difference was not significant (controls summer: 39.9 ± 11.7 ng/mL, controls winter: 37.8 ± 12.3 ng/mL, *P *= not significant). Hereafter, the summer levels of 25(OH)D_3 _in controls were used for comparison. There was vitamin D insufficiency (<30 ng/mL vitamin D level) in 41.6% of UCTD patients (67 patients) during the summer months, in 54.3% of patients (88 patients) during the winter, and in 18.64% of controls (11 subjects) (Table 1). In UCTD patients with vitamin D insufficiency, the winter levels of vitamin D were significantly lower than the summer levels (UCTD [<30 ng/mL] summer: 21.9 ± 4.7 ng/mL and winter: 18.1 ± 5.9 ng/mL, *P *= 0.03). Vitamin D deficiency (<10 ng/mL vitamin D level) was found in 5 of the UCTD cases.

**Table 1:** Seasonal fluctuation of the levels of vitamin D in patients with undifferentiated connective tissue disease

	UCTD patients Summer n = 161	UCTD patients Winter n = 161	Control Summer n = 59
Vitamin D insufficiency (<30 ng/mL)	67 patients (41.6%) 21.0 ± 5.79^a^	88 patients (54.3%) 18.4 ± 6.7^b^	11 (18.64%) 25.0 ± 4.65^c^

Vitamin D deficiency (<10 ng/mL)	5 (3.1%) (1 RA, 4 UCTD)	5 (3.1%) (2 RA, 3 UCTD)	

Significance: ^a-c^summer-control: *P *= 0.14; ^b, c^winter-control: *P *= 0.016; ^a, b^summer-winter: *P *= 0.03. RA, rheumatoid arthritis; UCTD, undifferentiated connective tissue disease.

### Correlation of clinical and laboratory parameters with plasma levels of vitamin D

**Figure 2:**
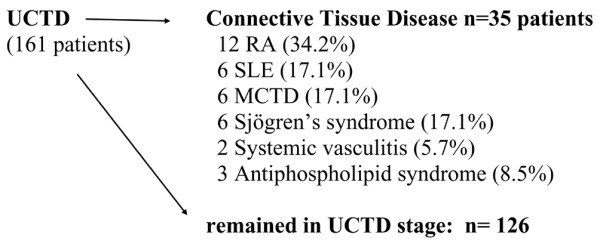
Evolution of undifferentiated connective disease to defined connective tissue diseases. MCTD, mixed connective tissue disease; RA, rheumatoid arthritis; SLE, systemic lupus erythematosus; UCTD, undifferentiated connective tissue disease.

Dermatological symptoms (photosensitivity, erythema, and discoid skin lesions) (*P *= 0.0046) and pleuritis (*P *= 0.0346) were also more frequent in UCTD patients with low levels of vitamin D (<30 ng/mL) (Table 2). Interestingly, patients with high serum levels of anti-U1-RNP, anti-SSA, and anti-CCP antibodies were found to have the lowest vitamin D levels (anti-U1-RNP: *P *= 0.024, anti-SSA: *P *= 0.029, and anti-CCP: *P *= 0.0001). During the follow-up period, 35 out of 161 UCTD patients (21.7%) developed an established CTD (Figure 2). The evolution to defined CTD was an average of 2.3 ± 1.2 years. Among these patients, 12 developed RA, 6 SLE, 6 MCTD, 6 Sjögren syndrome, 2 systemic vasculitis, and 3 antiphospholipid syndrome. Surprisingly, we found the lowest significant levels of vitamin D in those patients who eventually developed CTD compared with patients who remained in the UCTD stage (established CTD patients: 14.7 ± 6.45 ng/mL, remained in the UCTD stage: 33.0 ± 13.4 ng/mL, *P *= 0.0001) (Table 3).

**Table 2:** Relationship between low serum levels of vitamin D and clinical/serological manifestations of undifferentiated connective tissue disease patients

Correlation between clinical symptoms and vitamin D insufficiency	*P *value	OD (95% CI)
Xerostomia/xerophtalmia	0.8276	0.8909 (0.3768–2.107)
Polyarthritis	0.4834	0.7621 (0.3772–1.540)
Skin lesion	0.0046	2.987 (1.397–6.385)
Pleuritis	0.0346	5.367 (1.078–26.719)
Central and peripheral nervous system manifestations	1	0.8344 (0.1924–3.619)
Raynaud phenomenon	0.5331	0.7407 (0.3178–1.726)
Oesophageal involvement	0.3448	1.650 (0.6569–4.144)

Correlation between antibodies and vitamin D insufficiency		

Anti-U1-ribonucleoprotein antibody	0.0240	5.083 (1.286–20.094)
Anti-SSA antibody	0.029	3.474 (1.147–10.520)
Anti-cyclic citrullinated peptide antibody	0.0001	12.0 (3.541–42.036)
Anti-double-stranded DNA antibody	0.2551	0.4896 (0.1751–1.369)
Anti-neutrophil cytoplasmic antibody	0.2978	0.4024 (0.064–2.51)
Anti-cardiolipin antibody	0.3728	1.715 (0.6535–4.501)
Anti-SSB antibody	0.2945	0.5254 (0.1484–1.860)

OD, Odds ratio; CI, confidence interval

**Table 3:** Comparison of 35 patients who developed established connective tissue disease with 126 patients who remained in the stable stage of undifferentiated connective tissue disease

	Patients with evolution into defined CTD n = 35	Patients with 'stable' UCTD n = 126	*P *value
Age, years (mean ± standard deviation)	43.85 ± 11.1 range: 21–67	44.9 ± 12.7 range: 17–78	0.651

Duration of follow-up, years	2.31 ± 1.2 range: 0.5–4	4.09 ± 2.36 range: 0.5–9	0.0006113

Vitamin D serum levels, ng/mL	14.7 ± 6.45 range: 4.7–25.2	33.0 ± 13.4 range: 6–88.9	0.0001

CTD, connective tissue disease; UCTD, undifferentiated connective tissue disease.

## Discussion

Epidemiological studies suggest that the development of systemic autoimmune disease is affected by geographical areas and lifestyle. Presumably, in these processes, vitamin D is a significant environmental factor. Vitamin D deficiency has been linked to several different diseases, including malignant and immune-pathogenetic disorders. Age, gender, lifestyle, geographical areas, sunlight, and vitamin D supplementation are important determinants of vitamin D levels. In countries with temperate climates, such as Hungary, serum vitamin D concentrations rise and fall throughout each year in parallel with sun exposure [30-33]. The prevalence of vitamin D deficiency is much higher in Europe than in Asia, Australia, or the US. In Hungary, a high prevalence of hypovitaminosis D in healthy postmenopausal women has been described [34].

In the present study, we first analyzed the circulating levels and seasonal variance in the levels of 25(OH)D_3 _in a large cohort of patients with UCTD. According to our studies, in UCTD patients, vitamin D levels were significantly lower than in the control group both during the summer and winter months. Circulating levels of vitamin D fluctuate seasonally in UCTD patients, with low levels of 25(OH)D_3 _in the winter months and high levels during the summer months. Our data suggested that patients with UCTD have vitamin D insufficiency in 41% of cases in the summer months and even more became vitamin D-deficient during the wintertime. In UCTD patients, the winter levels of vitamin D were considerably lower than the summer levels. Plasma levels of 25(OH)D_3 _in UCTD patients were significantly lower compared with controls both in summer and winter periods. Vitamin D deficiency was found in 5 of the UCTD cases (3.1%) compared with none in the control group.

A clear correlation between the frequency of IDDM, MS, RA, SLE, and inflammatory bowel disease (IBD) and the north-south latitude, sunshine exposure, and vitamin D levels has been shown [12,35-37]. MS and IBD are diseases prevalent in Canada, the northern parts of the US, and Europe. The severity of MS has been shown to fluctuate seasonally, with exacerbations occurring mostly during the springtime [38,39]. Munger and colleagues [40] found that the risk of MS was 40% lower in women taking more vitamin D. This condition is explained by the fact that the northern hemisphere receives less sunlight, especially during the winter. MS, IDDM, and RA are more prevalent in temperate high latitudes than at the equatorial latitude. It seems that high vitamin D intake, regardless of sunlight exposure, is associated with a reduced risk of developing IDDM, RA, and MS. A study on 29,000 women showed that vitamin D intake reduced the risk of developing RA [41]. Vitamin D supplementation (2,000 IU/day) during infancy also significantly reduced the subsequent development of IDDM, evaluated 30 years later [12]. Vitamin D deficiency is common in patients with Crohn disease even when the disease is in remission [42].

25(OH)D_3_, 1,25(OH)_2_D_3_, and PTH levels in 25 Caucasian SLE patients (disease duration of 1 to 8 years) and in 25 female patients with fibromyalgia were studied, and no significant difference between the two groups was found [43]. Müller and colleagues [44] assessed the levels of 25(OH)D_3 _and 1,25(OH)_2_D_3 _in 21 SLE patients, 29 RA patients, and 12 osteoarthritis patients and found that vitamin D levels in SLE patients were significantly lower compared with patients with osteoarthritis and controls [44]. Significantly lower serum 25(OH)D_3 _levels were found among 123 recently diagnosed SLE patients compared with 240 age- and gender-matched controls from the Carolina Lupus Study [13]. Levels of 25(OH)D_3 _were significantly lower among African-Americans compared with Caucasians. Levels of vitamin D were highest in the summer and lowest in the winter. Vitamin D deficiency was found in 18% of the SLE patients with the presence of severe renal disease and photosensitivity [13].

In our results, the probability to develop dermatological symptoms (photosensitivity, vasculitis, and erythema) and pleuritis correlated with vitamin D insufficiency. The presence of anti-U1-RNP, anti-SSA, and anti-CCP occurred more frequently in these particular patients.

Data in our study as well as those reported by others suggest that UCTD may develop into any well-defined CTDs [16-18]. Evolution into a specific established CTD was found in 21.7% of patients with UCTD during the follow-up period. UCTD most frequently progressed into RA, and SLE, Sjögren syndrome, and MCTD had about the same prevalence. Interestingly, the lowest levels of vitamin D (<30 ng/mL) were measured in UCTD patients who subsequently evolved to defined CTDs. In our previous study, we found the shift toward Th1 with increased IFN-γ production in patients with UCTD combined with the degree of immunoregulatory disturbances characterized by the progressive divergent shifts in natural and induced T-regulatory cell populations [45]. Therefore, immunoregulatory abnormality signifies the transition from undifferentiated to definitive CTD [45]. Since vitamin D is an important regulator of the immune system, it raises the possibility that vitamin D deficiency may contribute to the progression into well-defined CTDs.

Several factors can lead to low levels of vitamin D in our patients with UCTD. Although the physical activity of most patients was not limited, patients with photosensitive rashes do seem to have a reduced exposure to sunlight and generally use very high UV protection. As another vitamin D-reducing factor, anti-vitamin D antibodies have been described in patients with SLE, antiphospholipid syndrome, and pemphigus vulgaris, and these autoantibodies were associated with anti-dsDNA antibodies in SLE [46].

The observed low vitamin D levels underline the importance of an intensified routine vitamin D supplementation as opposed to the current administration practice. This is further supported by a few prospective studies showing that the intake of vitamin D significantly reduces the incidence and/or progression of autoimmune diseases [40,47,48].

Based on our findings, we conclude that the measurement of serum vitamin D is crucial in UCTD patients and that the effective supplementation of vitamin D may be important in these patients. Future prospective studies are needed to determine the efficacy of supplementation of vitamin D in the prevention of the subsequent evolution of UCTD to well-defined CTDs and to establish the role of vitamin D in the treatment of autoimmune diseases.

## Conclusions

Vitamin D has a pivotal role in the maintenance of immune homeostasis. In various systemic autoimmune diseases, low levels of vitamin D have been described previously. We showed that, in patients with UCTD, serum levels of vitamin D were significantly lower compared with healthy individuals. Moreover, critically low levels of the vitamin clearly correlated with the progression to well-established CTDs. Our findings support the idea that vitamin D may be a key regulator of autoimmune processes in patients with UCTD.

## Abbreviations

anti-CCP: anti-cyclic citrullinated peptide; anti-CL: anti-cardiolipin; anti-dsDNA: anti-double-stranded DNA; anti-U1-RNP: anti-U1-ribonucleoprotein; BMD: bone mineral density; CTD: connective tissue disease; ELISA: enzyme-linked immunoabsorbent assay; HPLC: high-performance liquid chromatograph; IBD: inflammatory bowel disease; IDDM: insulin-dependent diabetes mellitus; IFN-γ: interferon-gamma; IL: interleukin; MCTD: mixed connective tissue disease; MS: multiple sclerosis; PTH: parathyroid hormone; RA: rheumatoid arthritis; RF: rheumatoid factor; SLE: systemic lupus erythematosus; UCTD: undifferentiated connective tissue disease.

## Competing interests

The authors declare that they have no competing interests. The submission fee was sponsored in part by TEVA Hungary Ltd. (Budapest, Hungary).

## Authors' contributions

EZ performed acquisition and analysis of data. PS performed interpretation of data and manuscript preparation. JG, MZ, and GS performed interpretation of data and drafted the manuscript. JK, LC, and EG performed analysis and interpretation of data. EB gave final approval of the version to be published. All authors read and approved the final manuscript.
